# Artificial grassland establishment alters soil organic carbon fractions and *cbbL*-type carbon-sequestering microbial communities

**DOI:** 10.3389/fmicb.2025.1726039

**Published:** 2025-12-08

**Authors:** Linge Shan, Aasitaiken Julihaiti, Wenhao Wang, Die Lu, Yi Hu, Baolong Zhao, Yake He, Xiangkang Meng, Helong Yang

**Affiliations:** 1 Key Laboratory of Grassland Resources and Ecology of Xinjiang, College of Grassland Sciences, Xinjiang Agricultural University, Ürümqi, China; 2 Xinjiang Key Laboratory of Grassland Resources and Ecology, Ürümqi, China; 3 Key Laboratory of Grassland Resources and Ecology in Arid Desert Regions of Western China, Ministry of Education, Ürümqi, China

**Keywords:** artificial grassland establishment, particulate organic carbon (POC), mineral-associated organic carbon (MAOC), *cbbL* gene, carbon sequestration, microbial community

## Abstract

**Introduction:**

Soil organic carbon (SOC) dynamics and microbial drivers in arid regions are critical for ecosystem restoration and carbon sequestration. This study investigated how converting cropland to artificial grasslands influences SOC fractions and the community of carbon-fixing microorganisms in the arid region of Urumqi, Xinjiang, China.

**Methods:**

We established two types of artificial grasslands—grassy (GG) and leguminous (LG)—and compared them against adjacent cropland (CK). We analyzed SOC fractions, including particulate organic carbon (POC) and mineral-associated organic carbon (MAOC), and measured cumulative carbon mineralization. The abundance of the cbbL gene was quantified by quantitative PCR. The composition of the cbbL-harboring microbial community was characterized by sequencing the cbbL gene amplicons.

**Results:**

The MAOC content was significantly higher in GG than in CK and LG. Although the proportion of POC was higher in CK, its absolute content was lowest in LG. Cumulative carbon mineralization was significantly lower in CK than in GG and LG. The cbbL gene abundance was highest in LG. A total of 47,026 cbbL gene amplicon sequence variants were identified, predominantly from *Proteobacteria* and *Actinobacteria*, with facultative autotrophs as the dominant functional group. Compared to CK, both grassland types increased the relative abundance of *Actinobacteria* but decreased that of *Planctomycetes*. At the genus level, LG significantly enriched *SinoRhizobium* and *MesoRhizobium*, whereas GG promoted Microvirga and *Bradyrhizobium*.

**Discussion:**

Mantel tests identified soil pH, the proportions of soil aggregates (>2 mm, 2–0.25 mm, and <0.053 mm), electrical conductivity, and MAOC content as the key environmental factors shaping the carbon-fixing microbial community. These results demonstrate that the establishment of artificial grasslands, particularly leguminous types, enhances the potential for soil carbon sequestration by modulating key soil properties and enriching specific carbon-fixing microbial taxa. This study provides a scientific basis for using artificial grasslands to enhance ecological restoration and soil carbon sequestration in arid regions.

## 1 Introduction

Grasslands are a vital ecosystem type, covering approximately 40% of the Earth’s terrestrial area and representing 69% of land used for agricultural production (Duan et al., 2025). Against the backdrop of global warming and increasing anthropogenic influence, grassland degradation is widespread and continues to intensify (Chen H. et al., 2025; Chen X. et al., 2025; Chen Y. et al., 2025). Statistics indicate that about 50% of grasslands have experienced some degree of degradation and that arid and semi-arid regions are particularly affected (Yao et al., 2025; Shi et al., 2025). Grassland degradation can cause environmental problems, such as soil carbon loss, that significantly alter the stability of carbon pools in these ecosystems, ultimately contributing to the deterioration of the global carbon cycle and soil nutrient status (Wang X. et al., 2024; Wang Z. et al., 2024; Wang W. et al., 2024). Artificial grasslands are key to ecological restoration and agricultural intensification, and their establishment is associated with vegetation reconstruction and soil improvement, which can influence soil organic carbon (SOC) dynamics and microbial diversity. Efforts to increase agricultural production have resulted in the conversion of natural grasslands to cropland, which has resulted in the loss of soil organic matter, lower microbial diversity, and declining ecosystem services (Liu M. et al., 2020; Liu Y. et al., 2020). In contrast, the establishment of artificial grasslands can help mitigate soil degradation and increase carbon sequestration capacity, though the ecological effects vary with the phylogenetic identities and functional traits of planted species (She et al., 2024). The efficacy of vegetation restoration is directly relevant to the key regional concerns of productivity enhancement, ecological conservation, sustainable resource use, and economic development. Therefore, promoting the establishment of artificial grasslands and making full use of degraded grasslands can harmonize human activity and nature, ultimately promoting coordinated regional economic, ecological, and social development.

As a labile SOC fraction, particulate organic carbon (POC) serves as an indicator of short-term soil carbon sink potential (Dong et al., 2023), whereas the stability of mineral-associated organic carbon (MAOC) governs long-term carbon sequestration (Wang D. et al., 2025; Wang W. et al., 2025). Legumes can improve soil nitrogen availability via biological nitrogen fixation and can also promote the accumulation of POC, whereas the fibrous root systems of grasses may facilitate the stabilization of MAOC. Artificial grassland establishment can improve soil carbon sequestration by strengthening POC-MAOC coupling mechanisms. In contrast, agricultural land-use conversion and improper management practices may lead to the depletion of SOC fractions (Nyawasha et al., 2025).

As essential component of soil ecosystems, microorganisms play a key role in regulating the soil carbon cycle. Carbon fixed by microorganisms can be directly assimilated into microbial biomass, thereby facilitating soil carbon uptake and accumulation (Cao et al., 2023; Zhang D. et al., 2025; Zhang X. et al., 2025; Zhang Z. et al., 2025). Elucidating the factors that influence microbial carbon sequestration and their associated mechanisms is therefore of great importance for improving understanding of the soil carbon cycle. Carbon-fixing microorganisms employ various carbon fixation pathways to incorporate inorganic carbon into their own biomass, thereby transforming inorganic into organic carbon (Gan et al., 2024). Most photo- and chemoautotrophs fix CO_2_ via the Calvin cycle. The *cbbL* gene encodes ribulose-1,5-bisphosphate carboxylase/oxygenase (Rubisco), a key enzyme that catalyzes the first step of the Calvin cycle. As a result, the *cbbL* gene has been widely used to study the diversity of carbon-fixing bacteria in environmental samples, impacting a diversity of research ranging from aquatic ecosystems to photosynthetic bacteria to terrestrial ecosystems (He et al., 2024; Yin et al., 2022; Zhou et al., 2019). Currently, molecular ecological studies targeting the *cbbL* gene in carbon-fixing bacteria are predominantly conducted in dryland ecosystems (Yang et al., 2024). Such research is relevant for understanding how the establishment of artificial grasslands impacts different SOC fractions and carbon-fixing bacterial communities. It is also important for soil carbon pool management in arid regions, degraded ecosystem restoration, and sustainable agricultural development.

*Medicago sativa* and *Cynodon dactylon* are both high-quality perennial forage grasses that are planted widely throughout China. These species have unique effects on soil improvement, acting to fertilize the soil and maintain soil and water quality (Cai et al., 2023; Zhang D. et al., 2025; Zhang X. et al., 2025; Zhang Z. et al., 2025). This study was conducted in artificial grasslands (grasses + legumes) in the arid region of Urumqi, Xinjiang, China, using adjacent cropland as a control. Our objective was to examine how the establishment of grassy and leguminous artificial grasslands affects SOC components and the structure of carbon-fixing bacterial communities. We quantified *cbbL* abundances using high-throughput sequencing to assess the diversity and composition of carbon-fixing bacterial communities. These results were further integrated with measurements of soil physicochemical properties and SOC fractions to explore relationships between the structure of carbon-fixing microbial communities and environmental factors. Our findings provide a scientific basis for using assessments of microbial communities to guide soil carbon pool management in grassland ecosystems located in arid regions, thereby supporting practical strategies for enhancing soil carbon sequestration potential and sustainable management practices.

## 2 Materials and methods

### 2.1 Study area

The study area of this research is located in Sanping Internship Farm of Xinjiang Agricultural University, Toutunhe District, Urumqi City, Xinjiang (43°56′N, 87°35′E, elevation 580 m). This area, which was reclaimed in the 1950s, has a temperate continental semi-arid climate with abundant sunshine (2829.4 h), average annual precipitation of 228.80 mm, average annual evapotranspiration of 2,647 mm, a frost-free period of 163 d, an average annual temperature of 7.20 °C, and a maximum temperature of 42 °C. The soil type of the study area is mainly gravelly sandy loam. All soil in the experimental plots developed from the same parent material and has the same basic physicochemical properties. Sample plots were separated by 300–500 m.

### 2.2 Experimental design

In August 2023, three plots of *C. dactylon* and *M. sativa requires* grassland were selected separately. Additionally, three adjacent (approximately 100–300 m distant) cropland plots were chosen as controls. Before the grasslands were established, plots were managed in accordance with local agricultural conventions.

Within each of the three replicate plots per treatment (GG, LG, and CK), we collected three soil cores (0–20 cm depth) from adjacent sampling points (approximately 1–2 m apart) using a soil auger. Cores from the same plot were then thoroughly mixed to form one composite soil sample per plot. This composite sampling strategy was employed to average out small-scale spatial heterogeneity and obtain a representative microbial community profile for each treatment replicate, thereby enhancing the reliability of comparisons between treatments.

The *C. dactylon* grassland (GG) was established with the “Xinnong No. 2” cultivar, which was selected for its superior traits critical for survival in arid environments, including exceptional drought tolerance, strong cold resistance, and low maintenance requirements. These characteristics make it a highly representative and suitable choice for ecological restoration in northwest China.

The *C. dactylon* grassland (GG) consisted of the “Xinnong No. 2” *C. dactylon* germplasm resource nursery. Each plot covered an area of approximately 0.067 ha, with a spacing of approximately 100 meters between plots. Two *C. dactylon* plots were established in 2010, and one was established in 2013. Since establishment, the nursery has undergone only manual weeding and annual seed harvesting, with no mowing or fertilization. Irrigation was applied 2–3 times per year.

The *M. sativa* grassland (LG) was established with the “Xinmu No. 4” cultivar. This variety was chosen due to its pronounced tolerance to key environmental stresses in arid lands, particularly drought and soil salinity-alkalinity, ensuring its relevance and persistence for studying sustainable grassland establishment under local conditions.

The alfalfa grassland (LG) comprised the “Xinmu No. 4” *M. sativa requires* germplasm resource field, which was established in 2019. Each plot covers an area of approximately 0.067 ha, with plots separated by approximately 100–300 m. Since establishment, seeds have been harvested 1–2 times, and the *M. sativa* plots have been mowed 1–2 times annually, with no fertilization. Plots are irrigated 2–3 times a year.

The cropland control plots (CK) were established on farmland reclaimed from *Artemisia*-desert in the 1960s. From the 1960s to the 1990s, spring wheat and maize were the primary crops, transitioning mainly to tomato cultivation from the 1990s to the early 2020s. Since then, specialty fruit trees and other crops have been cultivated. The three sampled cropland plots each covered an area of approximately 0.133 ha, spaced approximately 200–400 meters apart. In the sampling year (2023), the respective crops grown were pumpkin, carrot, and tomato. Conventional farmland management practices were implemented and included annual tillage, basal fertilization, topdressing, and irrigation.

### 2.3 Soil sample collection

Soil samples (0–20 cm depth) were collected following a diagonal sampling pattern in August 2023. Within each plot, nine soil sampling points were established. Soil from three adjacent sampling points was pooled into one composite soil sample, resulting in three composite soil samples per plot. A total of 27 composite soil samples were collected, and transported to the laboratory following flash freezing using liquid nitrogen. Samples were divided into two parts: one part was air-dried for the determination of soil physicochemical properties, and the other part was stored at −80 °C for microbiological analyses.

### 2.4 Methods of analysis

#### 2.4.1 Determination of physical and chemical properties of soil

Soil pH was determined using the potentiometric method with a soil-water mass ratio of 1:2.5, and conductivity was determined using a conductivity meter. Soil total carbon (TC) was determined by the potassium dichromate oxidation-external heating method. Soil total nitrogen (TN) was determined by the Kjeldahl method.

#### 2.4.2 Determination of soil carbon fraction

“The separation of soil organic carbon into operational fractions of particulate organic carbon (POC) and mineral-associated organic carbon (MAOC) was performed according to the well-established particle-size-based physical fractionation procedure described by Cambardella and Elliott (1992). This method operationally defines the POC fraction as the organic matter retained on a 53-μm sieve after dispersion, and the MAOC fraction as the organic carbon associated with the material passing through the 53-μm sieve (Cambardella and Elliott, 1992). Briefly: 20 g (10 g) of soil samples were weighed and transferred to 250 mL triangular vials after 2 mm air drying process, and 60 mL of sodium hexametaphosphate (5 g·L^−1^) was added. After shaking by hand for 10–15 min, the sample was put on a reciprocating oscillator (18 °C, 90 rpm·min^−1^) for 18 h and the dispersed solution was passed through a 53 μm sieve and washed with pure water until the water under the sieve was clear, and the upper part of the sieve contained particles of organic matter, and was separated and dried in an aluminum box at 60 °C overnight (72 h), after which it was weighed. The lower portion of the sieve (<53 μm) was mineral-bound organic matter, which was evaporated in a water bath (90 °C) and then dried in an oven (24 h). Fractions were weighed and calculated as a percentage of whole soil. The >53 μm soil particles were ground and sieved through a 0.149 mm sieve, and a portion of the sample was used to determine its organic carbon content, which was multiplied by its percentage of the soil to calculate the content of particulate organic carbon (POC), and the difference between the content of soil organic carbon (SOC) and particulate organic carbon (POC) was used as an estimate of mineral-bound organic carbon (MAOC).

Consequently, MAOC content was calculated as the difference between the total soil organic carbon (SOC) and the measured POC content, consistent with the aforementioned convention.

Soil organic carbon mineralization was determined using the laboratory incubation–alkali absorption method, which primarily reflects the activity of aerobic microbial respiration by quantifying CO₂ release. The procedure generally followed established methods (Dou et al., 2024). Fresh soil (50 ± 0.5 g) was placed in a 500 mL incubation bottle and pre-incubated at 25 °C for 3 days. A small tripod was placed inside the bottle, supporting a beaker containing 20 mL of 0.2 mol/L NaOH solution. The bottles were kept sealed throughout the incubation period. Aeration was performed twice daily for 30 min during the first week, once daily in the subsequent week, and once every two days thereafter. Two blank controls were included. The amount of CO₂ absorbed in the NaOH solution and the rate of organic carbon release were measured by hydrochloric acid titration on days 1, 2, 3, 4, 6, 9, 11, 16, 22, 28, and 30, but does not account for potential anaerobic pathways.

#### 2.4.3 Soil total DNA extraction

Total soil DNA was extracted using the Power soil DNAIsolation Kit (MOBIO, United States), and the procedure was carried out according to the manufacturer’s instructions. 2 μL of DNA solution was taken after DNA extraction to check the concentration and purity using an ultra-micro UV–visible spectrophotometer (Nanodrop ND1000, United States), and DNA integrity was detected by 1% agarose gel electrophoresis. After extraction, 2 μL of DNA solution was taken and tested for concentration and purity using an ultra-micro UV–Vis spectrophotometer (Nanodrop ND1000, United States), and DNA integrity was detected by 1% agarose gel electrophoresis.

#### 2.4.4 Amplification and sequencing *of cbbL*, a functional gene for soil carbon sequestration

The upstream primer for *cbbL* amplification was K2f (ACCAYCAAGCCSAAGCTSGG processing), and the downstream primer was V2r (GCCTTCSAGCTTGCCSACCRC), with fragment lengths from 492 to 495 bp. PCR reaction system (25 μL): 5 × Reaction Buffer buffer 5 μL, 5 × High GC Buffer buffer 5 μL, 2.5 mmol·L^−1^ dCKPs 2 μL, 1 μL each of forward and reverse primers, 0.25 μL of Q5 DNA polymerase, 2 μL of template DNA, ddH2O 8.75 μL. PCR reaction conditions: 98 °C pre-denaturation for 30 s, so that the template DNA was sufficiently denatured, and then amplification. PCR reaction conditions: pre-denaturation at 98 °C for 30 s to fully denature the template DNA, then amplification (98 °C for 15 s to denature the template, then cool to 50 °C for 30 s to anneal, and hold at 72 °C for 30 s to make the primer extend on the template to synthesize the DNA to complete a cycle), repeat the cycle 27 times to make the amplified DNA fragments accumulate in large quantities, and finally hold at 72 °C for 5 min to make the product extension complete and store at 4 °C. The PCR products were purified and sent to Nanjing Meiji Biological Company for double-end sequencing of community DNA fragments using the Illumina platform. The original test data were spliced, quality-controlled and optimized using the Vsearch method to obtain high-quality sequences, which were then clustered at 97% similarity level, and the representative sequences and ASVs were outputted to the Nucleotide Sequence Database (NSDB) for classification. The representative sequences of ASV were annotated by comparing with the CK database (Nucleotide Sequence Database), and the taxonomic identification results were obtained.

#### 2.4.5 Data processing and analysis

Basic data processing and statistical analysis were performed using Excel 2010 and SPSS 19.0. One-way analysis of variance (ANOVA) was conducted to examine the effects of artificial grassland establishment on soil physicochemical properties (e.g., pH, electrical conductivity, total carbon, total nitrogen), soil organic carbon fractions (particulate and mineral-associated organic carbon), organic carbon mineralization rate, abundance of the *cbbL* gene, and composition of the carbon-fixing microbial community (relative abundance at phylum and genus levels). Least significant difference (LSD) *post-hoc* tests (*α* = 0.05) were applied for multiple comparisons among sampling sites, aiming to determine whether significant differences existed in these key indicators after grassland establishment and to provide a basis for further analysis of soil carbon pools and carbon-fixing microorganisms.

Quantitative abundance profiles of the *cbbL* gene were visualized using Origin 2024 to clearly display differences in gene abundance under artificial grassland treatments and illustrate variations in carbon sequestration potential at the genetic level. Microbial community composition at the phylum and genus levels was analyzed using QIIME2 (version 2019.4) to characterize the community structure of carbon-fixing microorganisms and identify dominant taxa and their distribution patterns.

Beta-diversity was assessed using principal coordinate analysis (PCoA), a dimensionality reduction technique that visually represents similarities and dissimilarities in microbial community structure across treatments. The analysis was performed based on the Bray–Curtis dissimilarity matrix calculated from the ASV-level abundance table, using the “ape” package in R. To statistically evaluate the significance of community structure differences observed in the PCoA plot, a permutational multivariate analysis of variance (PERMANOVA) was conducted. This test was implemented using the adonis2 function in the R package “vegan” with 999 permutations, as it is specifically designed for testing hypotheses about community data without relying on assumptions of normality. Furthermore, to identify specific ASVs that exhibited differential abundance across the treatment groups, an analysis of composition of microbiomes (ANCOM-BC) was applied. This method was employed using the ANCOMBC package in R due to its robustness in handling the compositionality and sparsity of sequencing data. Relationships between microbial community structure (based on the Bray–Curtis distance matrix), soil properties, and organic carbon components were examined using Mantel tests. This analysis aided in identifying key environmental drivers influencing microbial assembly.

All statistical analyses and visualizations, were generated using R and Origin 2024. The “ggplot2” package in R was primarily used for figure generation to ensure high visual clarity.

## 3 Results and analysis

### 3.1 Effects of artificial grassland establishment on soil physicochemical properties

Conversion to artificial grasslands significantly altered soil physicochemical properties (Table 1). Soil pH in GG was significantly higher than in the other two land-use types (*p* < 0.05), whereas soil conductivity was significantly lower (*p* < 0.05). Soil aggregates under different land uses were dominated by 2–0.25 mm particle size. Soil aggregates with particle sizes of 0.2 mm and 2–0.25 mm were significantly larger in GG than in CK and significantly larger in CK than LG (*p* < 0.05). Soil aggregates with 0.25–0.053 mm and <0.053 mm particle sizes were significantly larger in LG and higher than CK and GG (*p* < 0.05); soil total nitrogen content was the highest in CK, followed by GG and then LG (*p* < 0.05). Total carbon content was significantly higher in GG compared to CK and LG (*p* < 0.05).

**Table 1:** Soil physicochemical properties under GG, LG and CK treatments.

Treatment	pH	EC	>2 mm	2–0.25 mm	0.25–0.053 mm	<0.053 mm	TN (g/kg)	TC (g/kg)
GG	8.76 ± 0.13a	113.51 ± 5.29b	2.40 ± 0.19a	84.86 ± 1.54a	5.43 ± 0.74c	0.95 ± 0.26c	2.7 ± 0.07ab	20.2 ± 0.39a
LG	8.54 ± 0.09b	142.39 ± 7.21a	0.44 ± 0.14c	84.76 ± 0.96b	6.44 ± 0.53b	4.15 ± 0.23a	2.1 ± 0.04b	15.0 ± 0.11b
CK	8.64 ± 0.22ab	146.24 ± 9.85a	1.63 ± 0.16b	81.36 ± 1.74b	9.19 ± 0.32a	2.76 ± 0.34b	3.0 ± 0.06a	14.1 ± 0.10b

GG, LG, and CK denote grassy artificial grassland, leguminous artificial grassland, and cropland control, respectively; pH indicates soil acidity and alkalinity, EC represents electrical conductivity, TN signifies total nitrogen, and TC represents total carbon; >2 mm, 2–0.25 mm, 0.25–0.053 mm, and <0.053 mm represent soil aggregate size fractions based on particle diameter. Different lowercase letters denote statistically significant differences among treatments (*p* < 0.05).

### 3.2 Effects of artificial grassland establishment on soil organic carbon fractions

#### 3.2.1 Effects of artificial grassland establishment on mineral and particulate organic carbon

**Figure 1:**
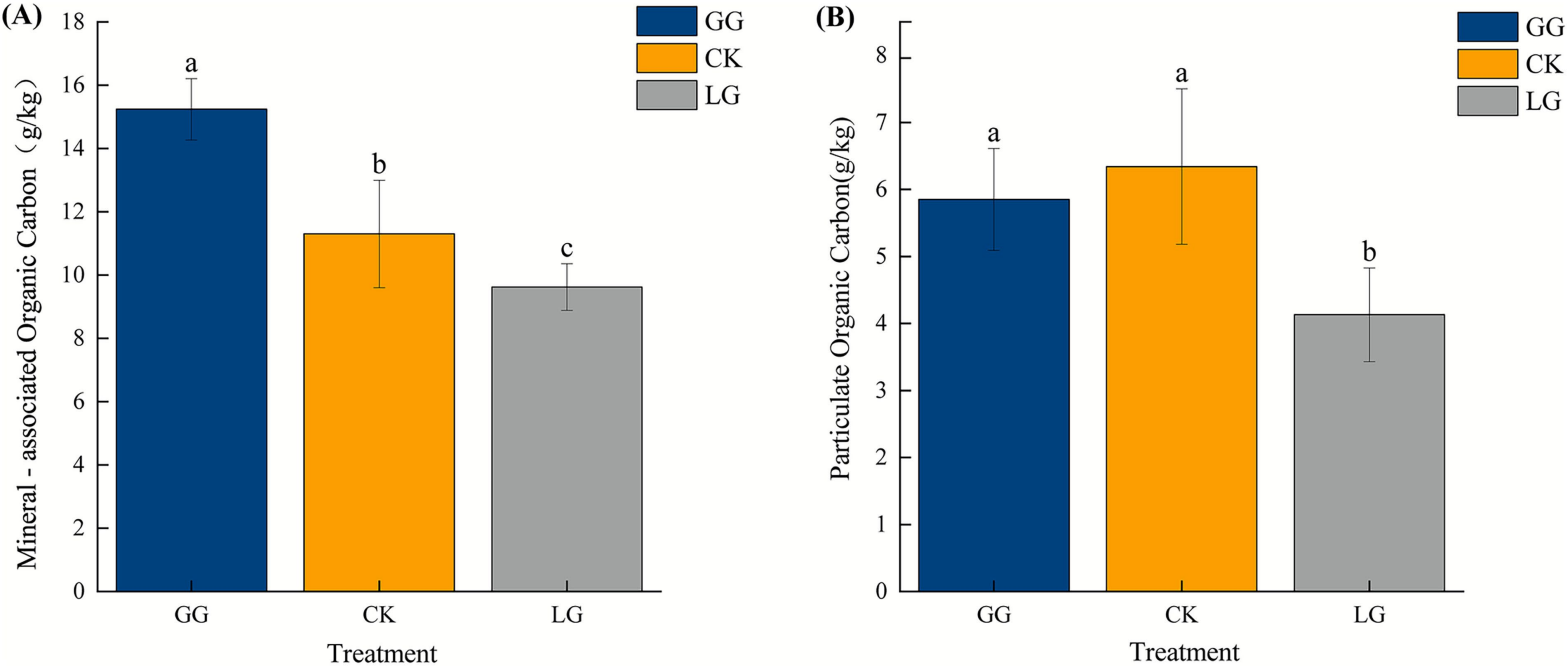
Mineral-associated organic carbon **(A)** and particulate organic carbon **(B)** contents under GG, LG, and CK treatments. GG denotes grassy artificial grassland, LG denotes leguminous artificial grassland, and CK denotes farmland control. Different lowercase letters denote statistically significant differences between treatments (*p* < 0.05).

Our analyses revealed that MAOC content was higher in GG than in CK or LG (*p* < 0.05) and significantly higher in CK than in LG (*p* < 0.05) (Figure 1A). POC content was significantly higher in CK than in GG or LG (*p* < 0.05), and POC content was lowest in LG (*p* < 0.05) (Figure 1B).

#### 3.2.2 Dynamics of soil organic carbon mineralization following artificial grassland establishment

**Figure 2:**
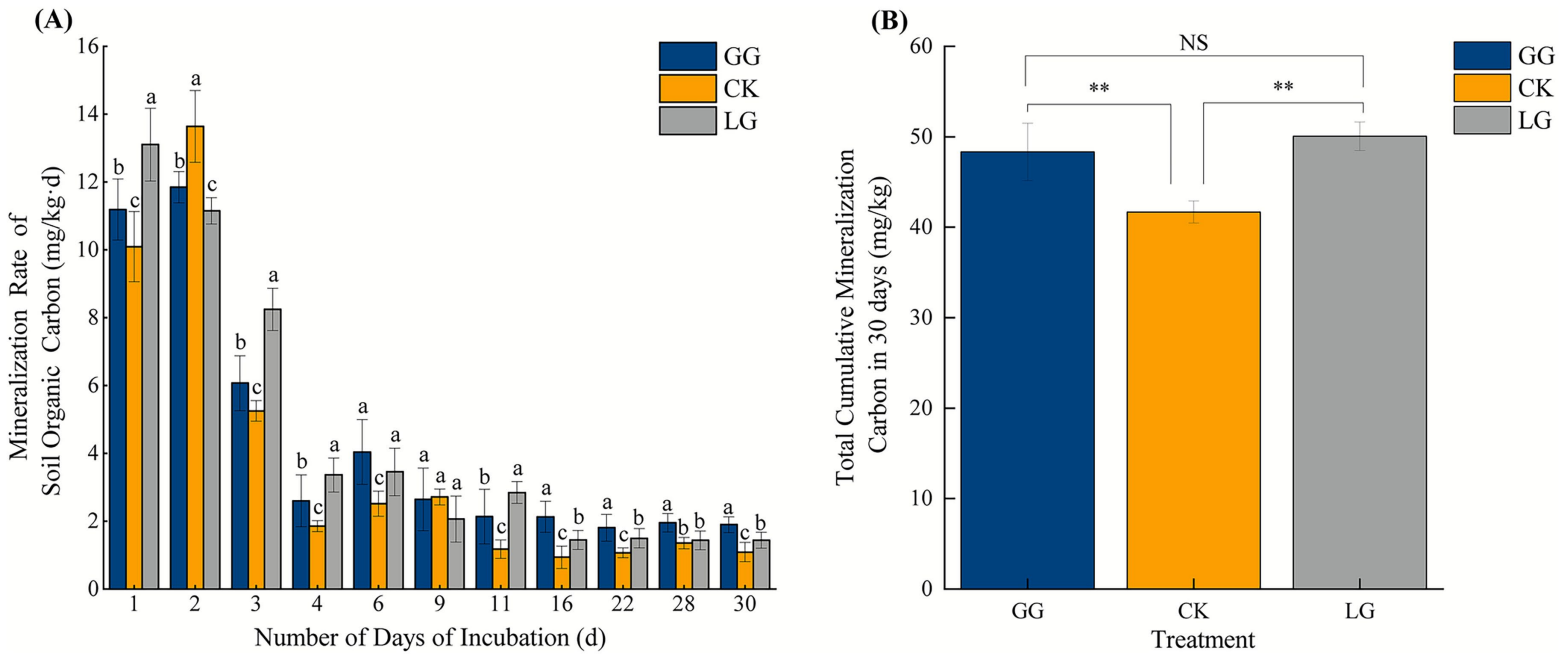
Soil organic carbon mineralization rate **(A)** and cumulative mineralized carbon **(B)** under different treatments following a 30-day incubation period. GG, LG, and CK represent grassy artificial grassland, leguminous artificial grassland, and cropland control, respectively. Different lowercase letters indicate statistically significant differences among treatments (*p* < 0.05). * and *** denote significant, highly significant, and extremely significant differences at *p* < 0.05, *p* < 0.01, and *p* < 0.001, respectively; NS indicates no significant difference (*p* > 0.05).

As shown in Figure 2, SOC mineralization rates during the incubation period initially increased before decreasing and stabilizing. Based on the rate of decline, the incubation period can be divided into three phases: the initial phase (days 1–3), during which mineralization was rapid and rates were highly variable across all land-use types; the middle phase (days 4–11), characterized by a gradual decrease in the rate of CO_2_ production; and the final phase (days 16–30), during which mineralization rates exhibited further declines before stabilizing. Throughout the incubation, the mineralization rate was higher in artificial grasslands (GG and LG) compared to the control (CK) (Figure 2A).

Our analyses revealed that the amount of carbon mineralized during the 30 day incubation period (cumulative mineralized carbon; CMC) was significantly lower in CK compared to GG and LG (*p* < 0.05), whereas we did not observe a significant difference between GG and LG (*p* > 0.05) (Figure 2B).

### 3.3 Differences in the community structure of *cbbL*-type carbon-fixing microorganisms under artificial grassland establishment

**Figure 3:**
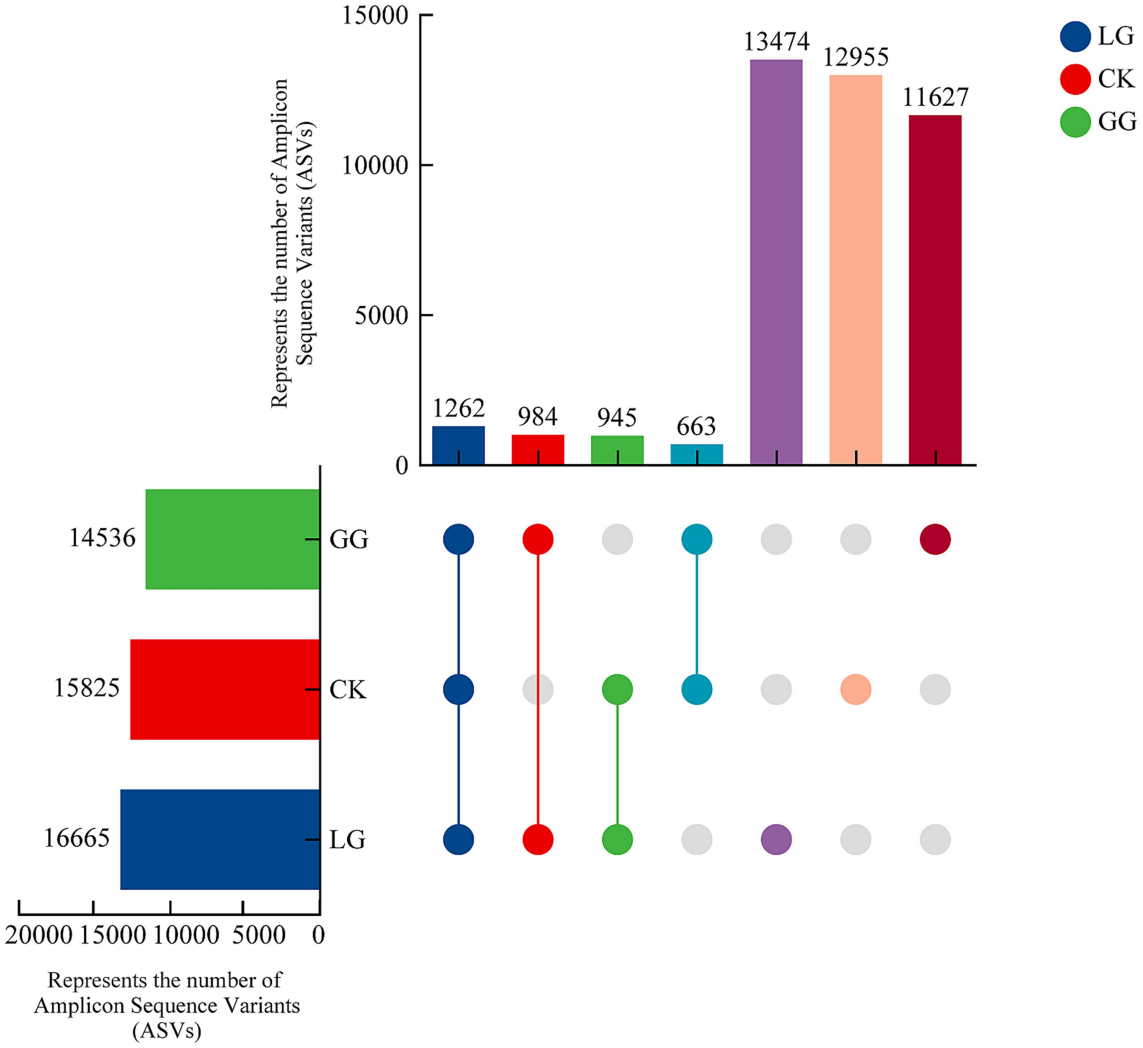
Shared and unique amplicon sequence variants (ASVs) of *cbbL*-type carbon-fixing microorganisms across GG, LG, and CK treatments. This UpSet plot illustrates the composition and overlap of ASVs among groups. The leftmost bar chart shows the total ASV count for each group (GG, CK, LG), with the *x*-axis representing ASV counts and the *y*-axis listing the groups (GG, CK, LG). In the central matrix, individual dots represent ASVs unique to a single group, and lines between dots denote shared ASVs between groups. The vertical bar chart above shows the number of ASVs in each intersection. GG, LG, and CK represent grassy artificial grassland, leguminous artificial grassland, and cropland control, respectively.

A total of 47,026 amplicon sequence variants (ASVs) were obtained in this study, with 12,955 identified in CK, 11,627 in GG, 13,474 in LG, and 1,262 shared among all three treatments. Additionally, 663 ASVs were shared between CK and GG, 945 were shared between CK and LG, and 984 were shared between GG and LG (Figure 3).

**Figure 4:**
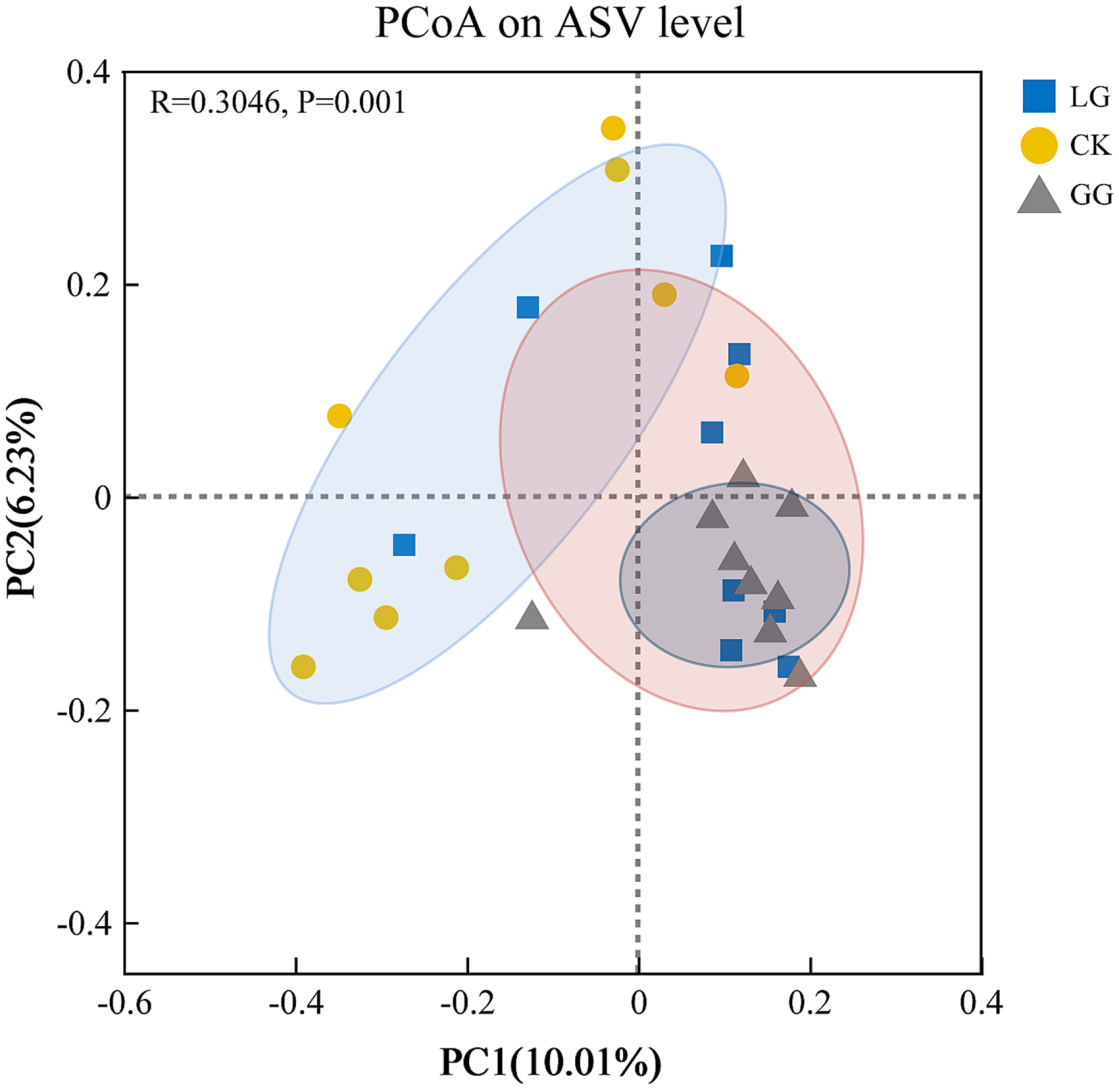
PCoA analysis of *cbbL*-type carbon sequestering microbial community structure under GG, LG and CK treatments. The horizontal and vertical axes represent the two selected principal coordinate components, with percentages indicating each component’s contribution to sample composition variation. The scale markings on both axes denote relative distances and have no intrinsic meaning. Points of different colors and shapes represent samples from distinct groups; closer proximity between two sample points indicates greater similarity in species composition between those samples. GG, LG, and CK represent grassy artificial grassland, leguminous artificial grassland, and cropland control, respectively.

To assess *β*-diversity, principal coordinate analysis (PCoA) was performed based on Bray–Curtis distances (Figure 4). The PERMANOVA test, recommended for community-level analysis, confirmed a statistically significant effect of the treatment group on the microbial community structure of *cbbL*-type carbon-fixing microorganisms (*F* = 1.517, *R*^2^ = 0.112, *p* = 0.001). This significant divergence was visually apparent in the PCoA plot, where the CK communities were primarily separated along PC1 (10.1% of variation), while GG and LG communities were differentiated along PC2 (6.23% of variation). The tighter clustering between GG and LG samples suggested a higher similarity in species composition, contrasting with the more distinct community structure of the CK group. This observation was further corroborated by the Analysis of Similarity (ANOSIM), which also indicated that between-group differences were significantly larger than within-group differences (*R* = 0.3046, *p* = 0.001).

### 3.4 Composition of *cbbL*-type carbon-fixing microbial communities under artificial grasslands

Community bar plots were generated to display the composition of soil samples across the three land-use types at the phylum and genus levels after removing taxa with an average relative abundance below 1% across all samples. In addition, given the broad taxonomic and functional diversity within Proteobacteria, this phylum was further visualized at the class level to better resolve its compositional variation among land-use types.

**Figure 5:**
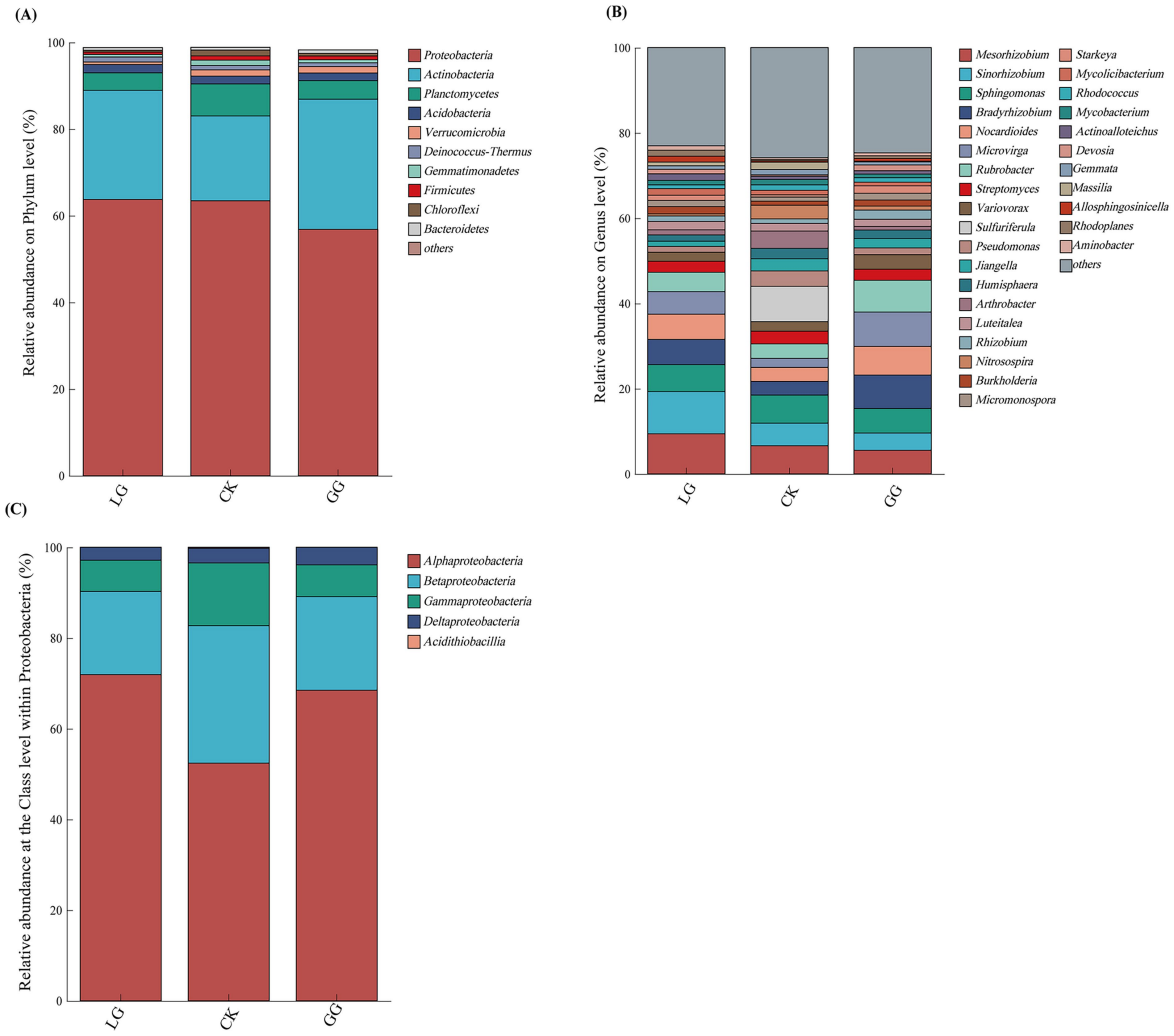
Community composition of *cbbL*-type carbon sequestering microorganisms under GG, LG and CK treatments. **(A)** Composition of microbial communities for gate-level carbon sequestration and carbon sequestration under different treatments. **(B)** Composition of microbial communities for horizontal carbon sequestration and carbon sequestration under different treatments. **(C)** Community composition of *cbbL*-type carbon sequestering microorganisms belonging to Proteobacteria, analyzed at the class level under different treatments. GG, LG, and CK represent grassy artificial grassland, leguminous artificial grassland, and cropland control, respectively.

Clear differences were observed in the composition of carbon-fixing bacterial communities across the different land-use types. At the phylum level (Figure 5A), the *cbbL*-type carbon-fixing communities were dominated by Proteobacteria (56.69–63.64%), Actinobacteria (19.5–29.95%), and Planctomycetes (4.31–7.41%), which together represented approximately 85% of the total community. Further class-level analysis of the Proteobacteria (Figure 5C) revealed a predominance of Alphaproteobacteria (52.39–71.92%) and Betaproteobacteria (18.35–30.33%), with comparatively lower contributions from Gammaproteobacteria (6.88–13.83%) and Deltaproteobacteria (2.85–3.88%).

At the genus level (Figure 5B), the most relatively abundant taxa in the CK treatment included Mesorhizobium (5.54–9.41%), Sinorhizobium (4.03–9.98%), and Sphingomonas (5.78–6.59%), among others. Notably, the dominant genera distinctly varied across treatments: the LG treatment was dominated by Sinorhizobium (9.8%) and Mesorhizobium (9.41%), the GG treatment was characterized by Microvirga (8.07%) and Bradyrhizobium (7.83%), and the CK community was primarily composed of Sulfuriferula (8.29%) and Sphingomonas (6.59%).

**Figure 6:**
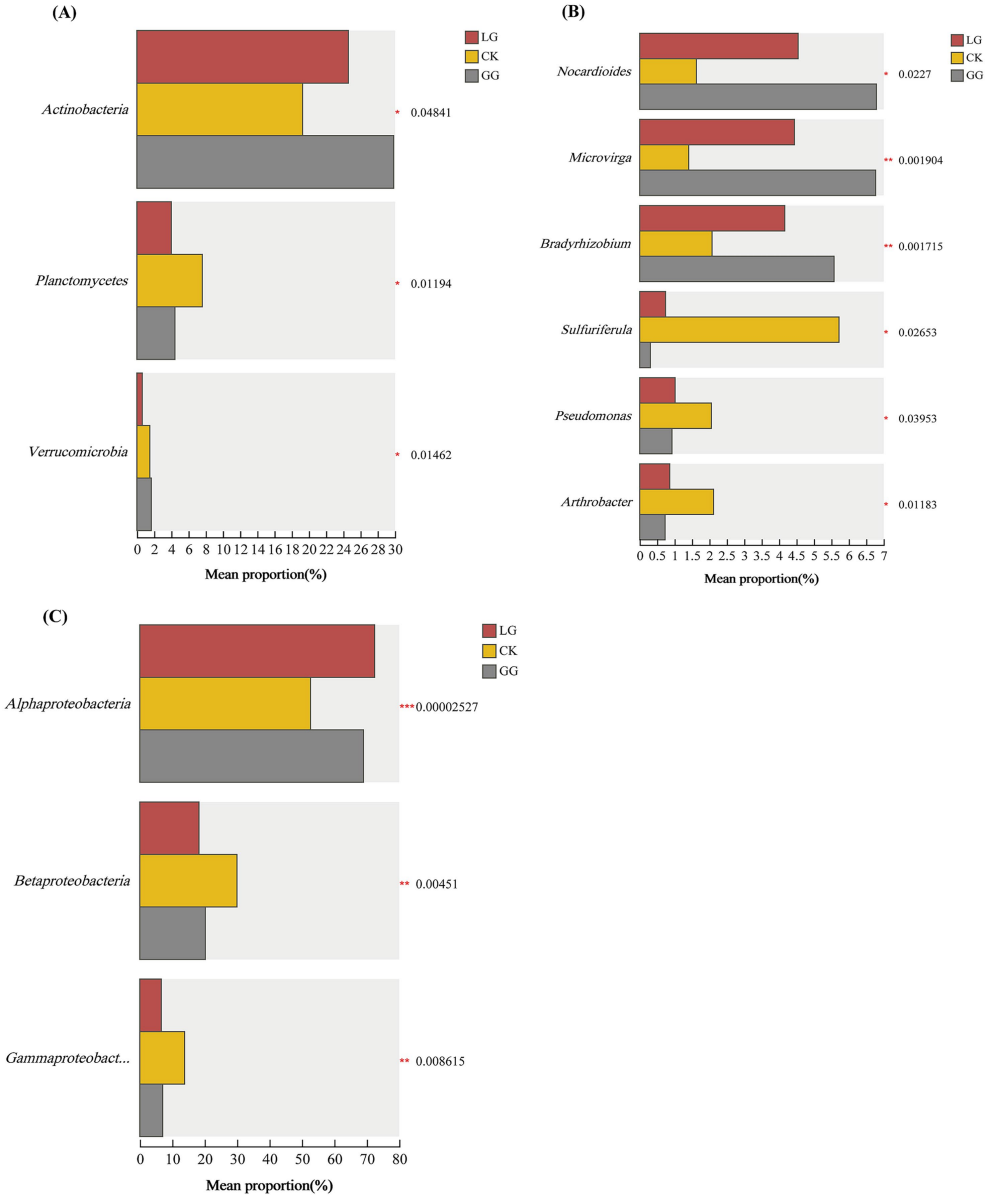
Analysis of significant flora differences in *cbbL*-type carbon sequestering microorganisms under GG, LG and CK treatments. **(A)** Significant flora differences of gate level carbon sequestering and carbon fixing microorganisms under different treatments. **(B)** Significant flora differences of sub level carbon sequestering and carbon fixing microorganisms under different treatments. **(C)** There are significant differences in the microbial communities involved in carbon sequestration and carbon fixation at the class level of the phylum *Proteobacteria* under different treatment conditions. * and *** indicate significant, highly significant, and extremely significant differences at *p* < 0.05, *p* < 0.01, and *p* < 0.001, respectively.

To rigorously identify differentially abundant taxa across land-use types, ANCOM-BC was applied to compare relative abundances of carbon-fixing bacteria. As shown in Figure 6A, the relative abundance of Actinobacteria was significantly higher in the GG treatment than in the LG or CK treatments (*p* < 0.05). The relative abundance of Planctomycetes was highest in the CK treatment (*p* < 0.01), while that of Verrucomicrobia was lowest in LG, intermediate in GG, and highest in CK, with significant differences among all treatments (*p* < 0.05; Figure 6A).

Within the Proteobacteria (Figure 6C), the relative abundance of Alphaproteobacteria was significantly lower in the CK treatment than in the GG and LG treatments (*p* < 0.001). In contrast, the relative abundances of Betaproteobacteria and Gammaproteobacteria were significantly higher in the CK treatment (*p* < 0.01).

At the genus level (Figure 6B), the relative abundances of Nocardioides, Microvirga, and Bradyrhizobium were significantly lower in the CK treatment than in the LG or GG treatments (*p* < 0.001). Conversely, the relative abundances of Sulfuriferula, Pseudomonas, and Arthrobacter were significantly higher in the CK treatment (*p* < 0.01).

### 3.5 Relationship between the community structure of *cbbL*-type carbon sequestering microorganisms and soil physicochemical properties in artificial grasslands

**Figure 7:**
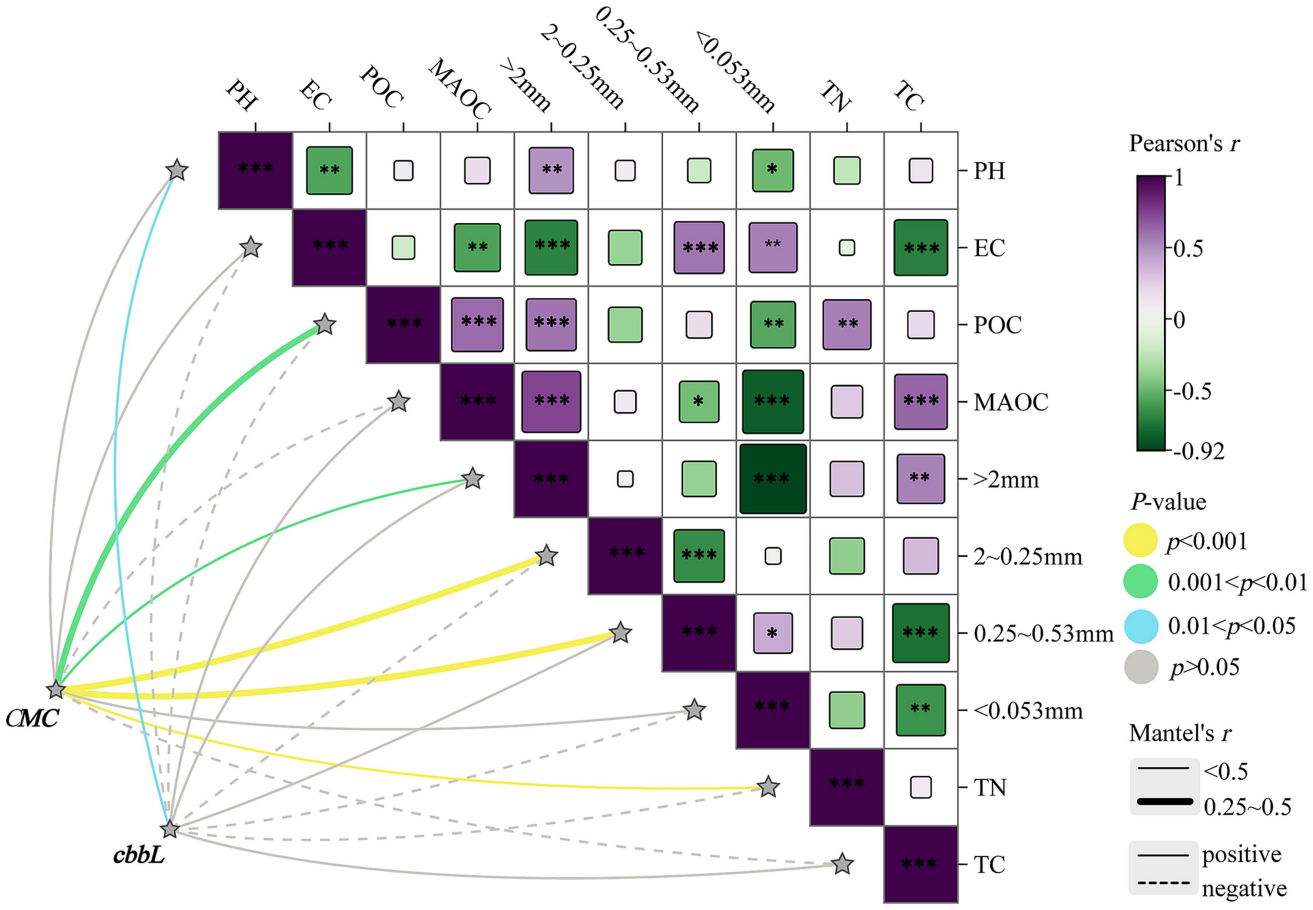
Mantel test analysis between total cumulative mineralized carbon, *cbbL*-type carbon-fixing microbial community structure, and environmental factors. GG, LG, and CK represent grassy artificial grassland, leguminous artificial grassland, and cropland control, respectively. EC denotes electrical conductivity, TN represents total nitrogen, and TC indicates total carbon; >2 mm, 2–0.25 mm, 0.25–0.053 mm, and <0.053 mm indicate soil aggregate size fractions; CMC refers to cumulative mineralized carbon of soil organic carbon, and *cbbL* represents the *cbbL*-type carbon-fixing microbial community. *, **, and *** denote significant, highly significant, and extremely significant differences at *p* < 0.05, *p* < 0.01, and *p* < 0.001, respectively.

Mantel test analysis was used to evaluate responses of *cbbL*-type carbon-fixing microbial communities to environmental factors. Results (Figure 7) indicated a significant positive correlation between microbial community structure and soil pH (*p* < 0.05). No significant correlations between community structure and other environmental factors were identified, suggesting that the community composition of *cbbL*-type carbon-fixing microorganisms was relatively stable and not strongly influenced by variation in soil properties across land-use types.

CMC was significantly and positively correlated with soil aggregates in the >2 mm, 2–0.25 mm, and <0.053 mm size classes (*p* < 0.001), indicating that soil aggregate formation and stability had a positive impact on microbial respiration. Significant positive correlations were also observed between electrical conductivity (EC) and MAOC (*p* < 0.01), suggesting that these factors play important roles in regulating soil microbial respiration. These findings further reveal the integrated influence of soil physicochemical properties on microbial community structure and respiratory activity (Figure 7).

**Figure 8:**
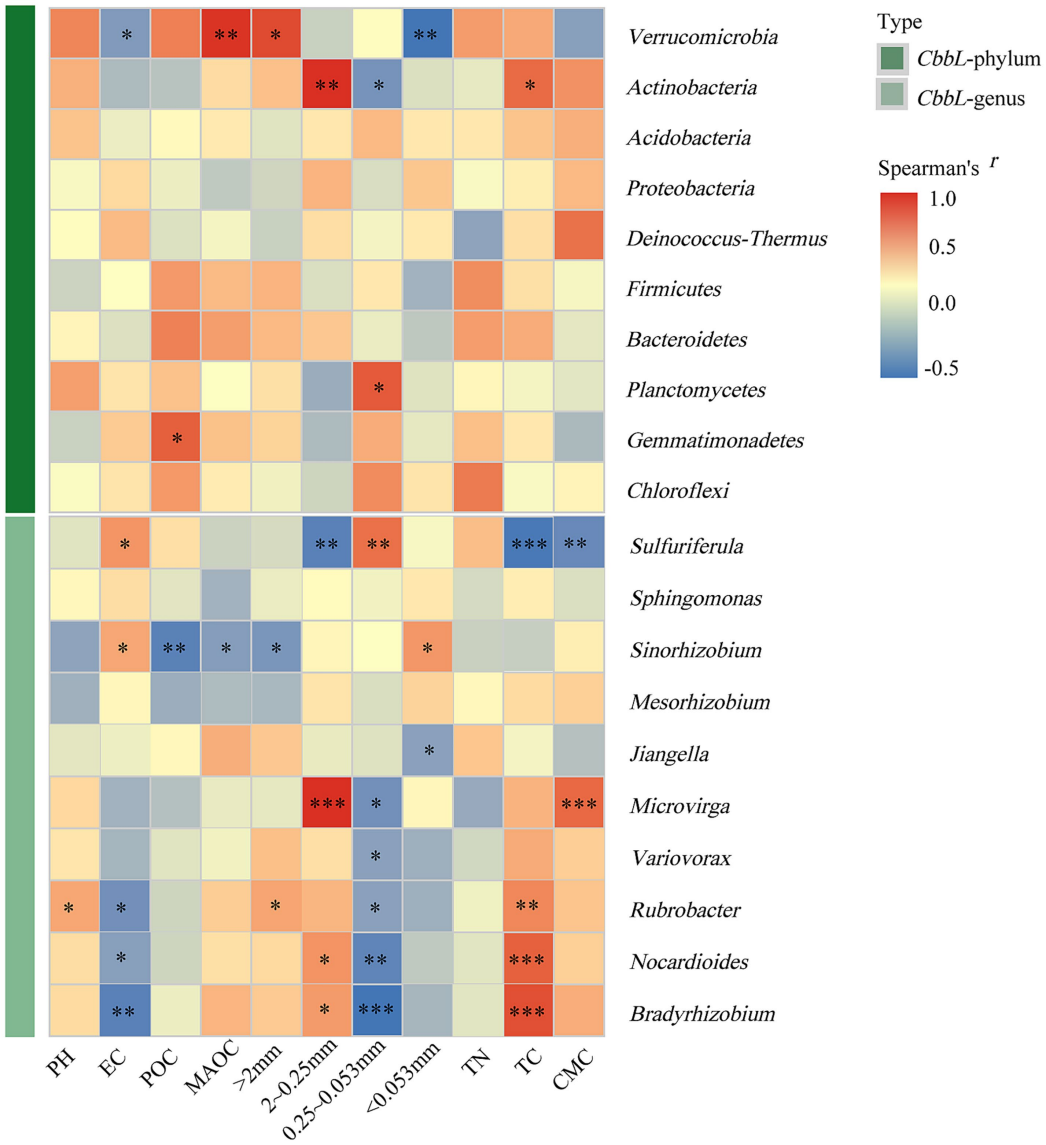
Spearman correlation analysis between environmental factors and *cbbL*-type carbon-fixing microorganisms. GG, LG, and CK represent grassy artificial grassland, leguminous artificial grassland, and cropland control, respectively. EC denotes electrical conductivity, TN represents total nitrogen, and TC indicates total carbon; >2 mm, 2–0.25 mm, 0.25–0.053 mm, and <0.053 mm correspond to soil aggregate size fractions; CMC refers to cumulative mineralized carbon of soil organic carbon. *, **, and *** denote significant, highly significant, and extremely significant differences at *p* < 0.05, *p* < 0.01, and *p* < 0.001, respectively.

Spearman correlation analysis further identified significant relationships between the 10 most abundant taxa within *cbbL*-type carbon-fixing microbial communities and environmental factors (Figure 8).

At the phylum level, Verrucomicrobia exhibited significant positive correlations with both the proportion of aggregates in the >2 mm size fraction and MAOC (*p* < 0.05 and *p* < 0.01, respectively), and a significant negative correlation with aggregates in the <0.053 mm size fraction (*p* < 0.01). *Actinobacteria* was positively correlated with aggregates in the 2–0.25 mm size fraction and total carbon (TC) (*p* < 0.05). *Planctomycetes* exhibited a significant positive correlation with aggregates in the 0.25–0.053 mm size fraction (*p* < 0.05), while *Gemmatimonadetes* was positively correlated with POC (*p* < 0.05) (Figure 8).

At the genus level, *Sulfuriferula* was positively correlated with EC and the proportion of aggregates in the 0.25–0.053 mm size fraction (*p* < 0.05), and negatively correlated with the proportion of aggregates in the 2–0.25 mm size fraction, RC, and CMC (*p* < 0.01). *SinoRhizobium* was significantly and negatively correlated with the proportion of aggregates in the >2 mm fraction, MAOC, and POC (*p* < 0.01), but positively correlated with EC and the fraction of aggregates in the <0.053 mm size fraction (*p* < 0.05). *Rubrobacter* was positively correlated with pH, the proportion of aggregates in the >2 mm size fraction, and TC (*p* < 0.05), and negatively correlated with EC and the proportion of aggregates in the 0.25–0.053 mm size fraction (*p* < 0.05). *Jiangella* was negatively correlated with the proportion of aggregates in the <0.053 mm size fraction (*p* < 0.05).

*Microvirga* was positively correlated with the proportion of aggregates in the 2–0.25 mm size fraction and PMC (*p* < 0.001) and negatively correlated with the proportion of aggregates in the 0.25–0.053 mm size fraction (*p* < 0.05). *Variovorax* showed a significant negative correlation with the proportion of aggregates in the 0.25–0.053 mm size fraction (*p* < 0.05). *Nocardioides* was positively correlated with the proportion of aggregates in the 2–0.25 mm size fraction (*p* < 0.05) and positively correlated with TC (*p* < 0.001), but negatively correlated with EC and the proportion of aggregates in the 0.25–0.053 mm size fraction (*p* < 0.05). *BradyRhizobium* exhibited significant positive correlations with the proportion of aggregates in the 2–0.25 mm size fraction (*p* < 0.05) and TC (*p* < 0.001), a significant, negative correlation with EC (*p* < 0.01), and a significant, positive correlation with the proportion of aggregates in the 0.25–0.053 mm size fraction (*p* < 0.001).

## 4 Discussion

### 4.1 Alterations in soil organic carbon fractions following artificial grassland establishment

Soil organic carbon (SOC) pools can be separated via particle size fractionation into particulate organic carbon (POC, 53–2,000 μm) and mineral-associated organic carbon (MAOC, <53 μm). POC plays a crucial role in stabilizing SOC and serves as an important substrate for microbial respiration and carbon flux, making it essential for long-term SOC sequestration (Yang et al., 2024; Zhang D. et al., 2025; Zhang X. et al., 2025; Zhang Z. et al., 2025). Critically, in the context of our study conducted in an arid region, the transformation and persistence of both POC and MAOC are predominantly governed by aerobic microbial processes. The coarse-textured, sandy loam soil, characterized by high porosity and weak aggregate structure, facilitates profound oxygen infiltration, sustaining active aerobic bacterial respiration even at the 20 cm depth (Neira et al., 2015). This environmental precondition ensures that the microbial mineralization of carbon, particularly the labile POC pool, is primarily an aerobic process (Han et al., 2025) thereby validating the methodological approach of our respiration assay.

This study revealed significant differences in both POC and MAOC content among the GG, LG, and CK groups. Higher MAOC in GG compared to CK suggests a higher capacity for carbon sequestration in grassy artificial grasslands. Furthermore, MAOC was also higher in GG compared to LG, which may be attributed to the longer establishment period and more developed root system in GG plots, which increase SOC accumulation and stabilization. Previous studies have indicated that leguminous alfalfa cultivation in croplands (such as CK) can increase POC in surface soil (Cai et al., 2023). In contrast, we measured the lowest POC content in LG. This may be attributed to the relatively short establishment period of the leguminous grassland, which likely limited the development of a well-formed root-soil aggregate architecture in the surface, an interpretation that is consistent with our analysis of aggregate size. Limited formation of such aggregates may have decreased the physical protection available for POC (Li C. et al., 2024; Li J. et al., 2024).

Measurements of CMC reflect microbial activity and SOC transformations (Jagadamma et al., 2014). Here, we observed lower CMC in CK compared to GG and LG, consistent with findings reported by MenGGun Liu et al. (Wei et al., 2024). This difference may be attributable to the more stable soil aggregate structure in artificial grasslands (Feng et al., 2022). Furthermore, Mantel test analysis revealed a significant positive correlation between soil aggregate size fractions and SOC mineralization rates (*p* < 0.05), suggesting that soil aggregate stability helps facilitate the transformation of mineralizable carbon, enhancing microbial activity and increasing mineralization rates.

### 4.2 Artificial grassland establishment significantly alters the diversity of soil *cbbL*-type carbon-fixing microbial communities

The *cbbL* gene, encoding the form I RuBisCO large subunit, is a key molecular marker for autotrophic microorganisms that fix CO₂ via the Calvin cycle. Its abundance provides a proxy for the genetic potential of microbial carbon sequestration in soils (Liang et al., 2025; Li C. et al., 2024; Li J. et al., 2024). This study revealed that the abundance of the *cbbL* gene followed the order: LG > CK > GG, with the leguminous grassland (LG) exhibiting the highest potential for microbial carbon fixation.

The significantly higher *cbbL* gene abundance under LG indicates a greater inherent capacity for converting atmospheric CO₂ into organic soil carbon. This pattern can be mechanistically explained by shifts in the microbial community structure. The LG soil was distinctly enriched with specific bacterial genera, most notably *SinoRhizobium* and *MesoRhizobium*. While renowned for their nitrogen-fixing symbiosis with legumes, many species within these genera are also facultative autotrophs that possess the *cbbL* gene and can fix carbon independently (Babalola et al., 2024). Therefore, the legume-driven proliferation of these specific taxa is a key microbial mechanism directly driving the observed higher *cbbL* gene abundance. This creates a community with a heightened potential for direct carbon assimilation, complementing the organic carbon inputs from plant litter.

The gene *cbbL*, which encodes the large subunit of RubisCO I, is frequently used as a molecular marker for carbon-fixing microorganisms, enabling extensive research on microbes associated with soil carbon sequestration across various ecosystems (Liang et al., 2025; Li C. et al., 2024; Li J. et al., 2024). The *cbbL* gene serves as a functional marker for carbon-fixing microorganisms, linking soil microbial community structure with genetic potential and thus improving understanding of microbial-environmental interactions (Derevenets et al., 2024; Liu M. et al., 2020; Liu Y. et al., 2020). The abundance and diversity of these microorganisms are influenced by factors such as land use type, elevation, and vegetation (Chen H. et al., 2025; Chen X. et al., 2025; Chen Y. et al., 2025; Zhao et al., 2021). This study found that the abundance of the *cbbL* gene decreased in the order: LG > CK > GG. Although the mechanisms underlying the effects of artificial grassland establishment on *cbbL*-type carbon-fixing microbial communities, numerous studies have reported variation in the diversity of soil carbon-fixing microbes under different land-use types (Wang D. et al., 2025; Wang W. et al., 2025; Zhou et al., 2022). Rhizobia, which enhance nitrogen fixation and potentially upregulate *cbbL* gene expression (Babalola et al., 2024), were present in the LG but not the CK treatment. Studies in grassland and forest ecosystems further indicate that rhizobia represent a major group within carbon-fixing microbial communities (Anand et al., 2025; Babalola et al., 2024). Thus, the abundance of rhizobia in the LG treatment may have stimulated microbial growth and increased diversity. In contrast, the abundance of the *cbbL* gene was lower in GG compared to CK, possibly because of differences in soil pH. Previous work by He et al. demonstrated a positive correlation between *cbbL* gene abundance and pH (He et al., 2025). This is consistent with the soil pH values observed in this study, which were highest in LG, followed by CK and GG, aligning with the distribution of *cbbL* gene abundance. These results suggest that soil acidity/alkalinity significantly influences the activity of carbon-fixing microorganisms, consistent with previous reports (Sachiko et al., 2009) and underscoring the key role of pH in regulating the community structure of carbon-fixing microbes.

Numerous studies have shown that carbon-fixing bacteria harboring the *cbbL* gene belong predominantly to the phyla *Proteobacteria*, *Actinobacteria*, Cyanobacteria, and Firmicutes, whose activities influence soil carbon cycling and sequestration efficiency (Liang et al., 2025; Qin et al., 2021; Wang et al., 2019; Wang X. et al., 2024; Wang Z. et al., 2024; Wang W. et al., 2024). In this study, the *cbbL*-type carbon-fixing microbial community was primarily composed of *Proteobacteria* and *Actinobacteria*. *Proteobacteria*, known for their broad ecological niche and high environmental adaptability (Wu et al., 2025), were highly abundant across all treatments (GG, LG, and CK), suggesting their active involvement in carbon and nitrogen cycling, a finding consistent with previous research (Sorochkina et al., 2024; Tan et al., 2025; Zhang et al., 2023).

The relative abundance of *Proteobacteria* was significantly higher in both GG and LG treatments compared to CK, aligning with reports that grass-dominated ecosystems are often enriched with *Proteobacteria*, while leguminous systems tend to be dominated by a mix of Firmicutes and *Proteobacteria* (Wei et al., 2025). More importantly, our class-level analysis revealed that the relative abundance of *Alphaproteobacteria*—a key taxonomic group involved in carbon cycling with numerous species possessing carbon fixation potential (Ma et al., 2024; Zhu et al., 2025; Wang et al., 2022)—was significantly higher in LG and GG than in CK. This indicates that artificial grasslands, particularly legume-based systems, possess greater carbon sequestration potential than croplands. In contrast, the CK treatment was characterized by a predominance of *Gammaproteobacteria*, consistent with prior findings (Yang et al., 2023), a pattern potentially attributed to the high sensitivity of *Gammaproteobacteria* to fertilization practices (Tambong and Ren, 2013). Consequently, the microbial community in CK likely reflects a state shaped by agricultural disturbance, primarily oriented toward heterotrophic decomposition rather than efficient direct carbon fixation.

From the perspective of microbial functional groups, the dominant genera identified were primarily facultative autotrophs capable of fixing CO₂ via the Calvin cycle (Dai et al., 2024). The prevalence of this metabolic type aligns with observations in tropical croplands and other agricultural soils (Xu et al., 2021; Zhong et al., 2020), suggesting it is a common adaptive trait in managed terrestrial ecosystems. The metabolic flexibility of facultative autotrophs—allowing them to utilize organic carbon while maintaining autotrophic capacity—is a key competitive advantage in fluctuating soil environments (Wang X. et al., 2024; Wang Z. et al., 2024; Wang W. et al., 2024) and may underpin the robustness of the soil carbon sequestration process (Ma et al., 2022).

Specifically, genera such as *BradyRhizobium*, *MesoRhizobium*, and *Nocardia* are known facultative autotrophs (Long et al., 2024). Compared to CK, the relative abundances of *BradyRhizobium* and *MesoRhizobium* were significantly higher in LG and GG. Furthermore, the relative abundances of *SinoRhizobium* and *MesoRhizobium* were higher in LG than in GG. It is well established that nitrogen-fixing rhizobia associated with leguminous plants primarily include *α-Proteobacteria* genera such as *Rhizobium*, *BradyRhizobium*, and *SinoRhizobium* (Fang et al., 2023; Joshi et al., 2025; Murphy, 2024), which is consistent with our results. The dominance of these multi-functional microbes, capable of simultaneous nitrogen fixation and carbon assimilation, provides a mechanistic explanation for the superior carbon sequestration potential observed in the LG treatment.

These results underscore the considerable influence of vegetation type on soil microbial communities, offering evidence that establishing artificial grasslands can optimize microbial composition and enhance soil carbon sequestration efficiency.

### 4.3 Environmental factors drive changes in carbon-fixing microbial communities

Numerous studies have shown that soil carbon-fixing microbial communities are highly sensitive to changes in soil properties and environmental conditions (Han et al., 2024; Philip et al., 2018). Factors such as vegetation type, SOC content, fertilization regime, light availability, and soil depth influence the microbial diversity and the abundance of the *cbbL* gene (Bai and Zhang, 2025; Lydia et al., 2023; Shanin et al., 2024; Wang et al., 2021; Yao et al., 2023). Among these, soil pH is a key factor shaping the composition and activities of carbon-fixing microbial communities (Cao et al., 2022), and serves as a strong predictor of organic carbon storage in grassland and forest soils (Zhang et al., 2024). In this study, Mantel test results identified soil pH as an important environmental factor influencing the structure of *cbbL*-type carbon-fixing microbial communities, consistent with previous findings (Hu D. et al., 2024; Hu L. et al., 2024). SOC mineralization rates reflect the proportion of carbon decomposed and released due to microbial metabolic activity and is a key indicator of soil microbial vitality (Luan et al., 2023). This study identified a significant positive correlation between CMC and the proportion of aggregates in the <0.053 mm size fraction, which contrasts with some previous reports (Wen et al., 2024). This discrepancy suggests that microbial activity within micro-aggregates may be mediated by unrecognized mechanisms, warranting further investigation. These results also imply that association with micro-aggregates may reduce SOC stability. Additionally, EC and MAOC content were significantly and positively correlated with SOC mineralization rate, aligning with earlier research (Xu et al., 2024). Together, these findings demonstrate that microbial respiratory activity is interactively shaped by soil aggregate structure, EC, and MAOC content. Future studies should focus on elucidating the microbial mechanisms distinctive to micro-aggregates to better understand their potential influence on soil carbon cycling.

Soil physicochemical properties and land-use type are among the most important factors influencing soil microbial communities. Properties such as pH, water content, organic matter, and total nitrogen affect microbial habitats, both directly and indirectly (Christopher et al., 2023; Derevenets et al., 2024; Guo et al., 2025; Guo et al., 2025; Han et al., 2025; Zhao et al., 2021, 2025). At the phylum level, dominant bacterial taxa were primarily influenced by aggregate size and TC. In this study, *Actinobacteria* was significantly and positively correlated with both TC and the 2–0.25 mm soil aggregate size fraction (*p* < 0.05). The relationship with soil aggregates is consistent with previous findings (Qi et al., 2025), although some studies have reported significant negative correlations between the relative abundance of *Actinobacteria* and TC (Zhang D. et al., 2025; Zhang X. et al., 2025; Zhang Z. et al., 2025). This discrepancy may be due to the strong adaptive capacity of *Actinobacteria* to nutrient-poor conditions, and the role of members of this taxon in decomposing plant and animal residues, thereby facilitating the breakdown and utilization of carbon, nitrogen, and phosphorus (Chen H. et al., 2025; Chen X. et al., 2025; Chen Y. et al., 2025). At the genus level, the most abundant taxa were mainly influenced by aggregate size fractions, TC, and EC, with a few dominant genera also significantly impacted by CMC. In this study, the 2–0.25 mm, 0.25–0.053 mm, and <0.053 mm soil aggregate fractions were significantly and positively correlated with the relative abundances of *Sulfuriferula*, *SinoRhizobium*, and *BradyRhizobium* (*p* < 0.05). This may be attributed to the fact that these genera belong to the phylum *Proteobacteria* (Liu et al., 2024), which tends to be prevalent across size fractions. Thus, their relative abundance is closely linked to aggregate size distribution, further highlighting the key role of aggregate structure in shaping microbial community composition.

The positive correlation between TC and the abundance of *BradyRhizobium* likely reflects this taxon’s classification as copiotrophic, meaning that they are capable of rapid growth in carbon-rich environments (Yao et al., 2024). We also identified a significant positive correlation between EC and the relative abundances of *Sulfuriferula* and *SinoRhizobium* (*p* < 0.05), consistent with previous findings (Sharma et al., 2025). This result suggests that EC may promote growth among these taxa by influencing the availability of soil ions. In contrast, we observed a significant negative correlation between EC and *BradyRhizobium*, which diverges from some earlier reports (Han et al., 2024). This may indicate a higher sensitivity of *BradyRhizobium* to elevated salinity, where increased EC could inhibit its growth, highlighting its unique response to environmental stressors (Yei et al., 2021). CMC was positively correlated with *Microvirga* and negatively correlated with *Sulfuriferula*. This aligns with studies linking that primarily link *Proteobacteria* to soil respiration under dryland conditions (Ali et al., 2020; Zhou et al., 2024), since both *Sulfuriferula* and *Microvirga* belong to this phylum. These results suggest that the influence of *Proteobacteria* on soil microbial respiration is diverse and may be regulated by organic substrate type and mineralization extent (Sun et al., 2018; Su et al., 2025).

## 5 Conclusion

1. MAOC content was significantly higher in GG compared to CK, whereas it was significantly lower in LG compared to CK. POC content was significantly higher in CK compared to LG. Additionally, CMC was lower in CK than in either GG or LG.
2. Regarding the carbon-fixing microbial community, the abundance of the *cbbL* gene was highest in LG, followed by CK and then GG.
3. Key environmental factors driving the carbon-fixing microbial community.

Soil pH was identified as the key environmental factor influencing the overall structure of the *cbbL*-type carbon-fixing microbial community, whereas we did not observe significant correlations between community structure and other tested environmental variables. This indicates a degree of structural stability and resistance to common environmental fluctuations. Soil aggregate composition, EC, and SOC fractions (MAOC, TC, and POC) together regulated microbial respiration and the distribution of key taxonomic groups. Distinct microbial taxa exhibited specific responses to these environmental factors.

In summary, the establishment of artificial grasslands significantly enhanced SOC mineralization rates, CMC, and the abundance of carbon-fixing microorganisms. Leguminous artificial grasslands (LG) demonstrated greater carbon sequestration potential than grassy artificial grasslands (GG). Therefore, priority should be given to leguminous species in the establishment of artificial grasslands to increase carbon sink capacity and ecosystem carbon retention. In cropland management, attention should be given to balancing the distribution of SOC fractions to maintain the functional integrity of the soil carbon pool and to ensure the health and stability of soil ecosystems.

## Data Availability

The original contributions presented in the study are publicly available. This data can be found here: PRJNA1374831 (NCBI BioProject).
